# Comparing Effects of Climate Warming, Fire, and Timber Harvesting on a Boreal Forest Landscape in Northeastern China

**DOI:** 10.1371/journal.pone.0059747

**Published:** 2013-04-01

**Authors:** Xiaona Li, Hong S. He, Zhiwei Wu, Yu Liang, Jeffrey E. Schneiderman

**Affiliations:** 1 State Key Laboratory of Forest and Soil Ecology, Institute of Applied Ecology, Chinese Academy of Sciences, Shenyang, People’s Republic of China; 2 School of Natural Resources, University of Missouri-Columbia, Columbia, Missouri, United States of America; University College London, United Kingdom

## Abstract

Forest management under a changing climate requires assessing the effects of climate warming and disturbance on the composition, age structure, and spatial patterns of tree species. We investigated these effects on a boreal forest in northeastern China using a factorial experimental design and simulation modeling. We used a spatially explicit forest landscape model (LANDIS) to evaluate the effects of three independent variables: climate (current and expected future), fire regime (current and increased fire), and timber harvesting (no harvest and legal harvest). Simulations indicate that this forested landscape would be significantly impacted under a changing climate. Climate warming would significantly increase the abundance of most trees, especially broadleaf species (aspen, poplar, and willow). However, climate warming would have less impact on the abundance of conifers, diversity of forest age structure, and variation in spatial landscape structure than burning and harvesting. Burning was the predominant influence in the abundance of conifers except larch and the abundance of trees in mid-stage. Harvesting impacts were greatest for the abundance of larch and birch, and the abundance of trees during establishment stage (1–40 years), early stage (41–80 years) and old- growth stage (>180 years). Disturbance by timber harvesting and burning may significantly alter forest ecosystem dynamics by increasing forest fragmentation and decreasing forest diversity. Results from the simulations provide insight into the long term management of this boreal forest.

## Introduction

Climate warming has pronounced effects on forests worldwide, particularly in the high latitudes of the boreal forest region. These effects have altered forest productivity [1], [2], forest composition [3], and natural disturbance regimes directly and indirectly [4]–[6], and are expected to continue and intensify in the future [7], [8].

Changes in annual and seasonal temperatures and precipitation have directly impacted forest growth rate [9], [10] and the establishment of native species and exotic species [11], [12]. These changes can also alter competitiveness relations among species [13]–[15] and lead to shifts in species distributions [16]–[18]. The resulting alterations in forest composition [3] and distribution are expected to affect the sequestration of carbon by forests at broad spatial scales [1], [2].

Climate warming indirectly impacts forest compositions and species’ distributional patterns through its effects on natural disturbances such as fires [4], [19]–[21]. In boreal forests, fire is a force that can influence forest succession and structure [22]. Both predictions and observations indicate that fire occurrence and area burned have been projected to increase with longer and warmer growing seasons [5], [23]–[27]. For instance, Stocks et al. [25] projected that the areal extent of extreme fire danger in Russia and Canada could greatly increase. Flannigan et al. [23] showed that the annual burned area in Canada could increase by 74–118% by the end of this century. Wotton et al. [27] similarly indicated that fire occurrence in the boreal forests of Canada could increase by 75–140% by year 2100. Soja et al. [5] assessed the current situation of boreal ecosystems as they relate to previous predictions of climate-induced ecological change, and indicated that the area burned both in Siberia and North America over recent decades has been steadily increasing. Liu et al. [28] projected that the mean fire occurrence density of a boreal forest in northeast China would increase by 30–230% under climate warming by 2100. Previous studies indicated the effects of increased fires on forest composition and forest productivity may equal or exceed the direct effects of climate warming in the boreal forest region [6], [29], [30]. For example, Schumacher and Bugmann [30] showed that fire was likely to become almost as important in shaping the forest landscape in the Swiss Alps as the direct effects of climate warming.

Timber harvest is one of the main anthropogenic disturbances to forests. Harvesting alters woody biomass accumulation, forest composition, and patterns of tree distribution across the landscape, and these effects may continue under a climate changing scenario [31], [32]. He et al. [32] estimated tree species response to forest harvesting and increased fire due to climate warming in northern Wisconsin forests, and indicated that forest harvesting accelerated the decline of northern hardwood and boreal tree species. Gustafson et al. [31] predicted global change effects on Siberian forests and found that harvesting effects on forest composition in boreal forests in Siberia were more significant than effects of climate warming.

Currently, there is increasing interest in exploring effects of climate warming, burning, and timber harvesting on forest landscapes because quantifying these effects can provide a basis for developing forest management policy under a changing climate. However, predicting the effects of climate warming, burning, and harvesting on forest landscapes is challenging because forests ecosystems involved complex interactions among forest successional trends, natural disturbances (including fire) and anthropogenic disturbances (including timber harvesting).

In this study, we employed a forest landscape model (LANDIS) to simulate forest landscape responses to climate warming, burning, and timber harvesting. LANDIS, a widely used forest landscape disturbance and succession model, independently simulates forest succession, natural disturbances and anthropogenic disturbances [33], [34]. LANDIS can incorporate the effects of climate warming on tree species and allow these effects to interact with landscape processes in the simulations [35]. The objectives of this study were to (1) quantify the management, disturbances, and species succession in a boreal forest landscape in northeastern China under a changing climate, (2) design a factorial experiment to assess the relative effects of climate warming as determined by species establishment probabilities and increased fire associated with climate warming, and current harvesting on forest species composition, age structure, and spatial pattern.

## Methods

### 1. Study Area

Our study area included Huzhong Forestry Bureau (770,432 ha) and Huzhong Natural Reserve (166,906 ha), located on the north side of the Great Xing’an Mountains in northeastern China, which covers nearly a million ha (122°39′30′’ to 124°21′00′’E and 52°25′00′’ to 51°14′40′’N) and is primarily a hilly mountainous region ranging from 450 to 1500 m in elevation. The climate is terrestrial monsoon with long and severe winters (mean January temperature −25.5°C) and short, mild summers (mean July temperature 18°C). Precipitation, which peaks in summer, is 420 mm annually and is unevenly distributed throughout the year.

**Figure 1:**
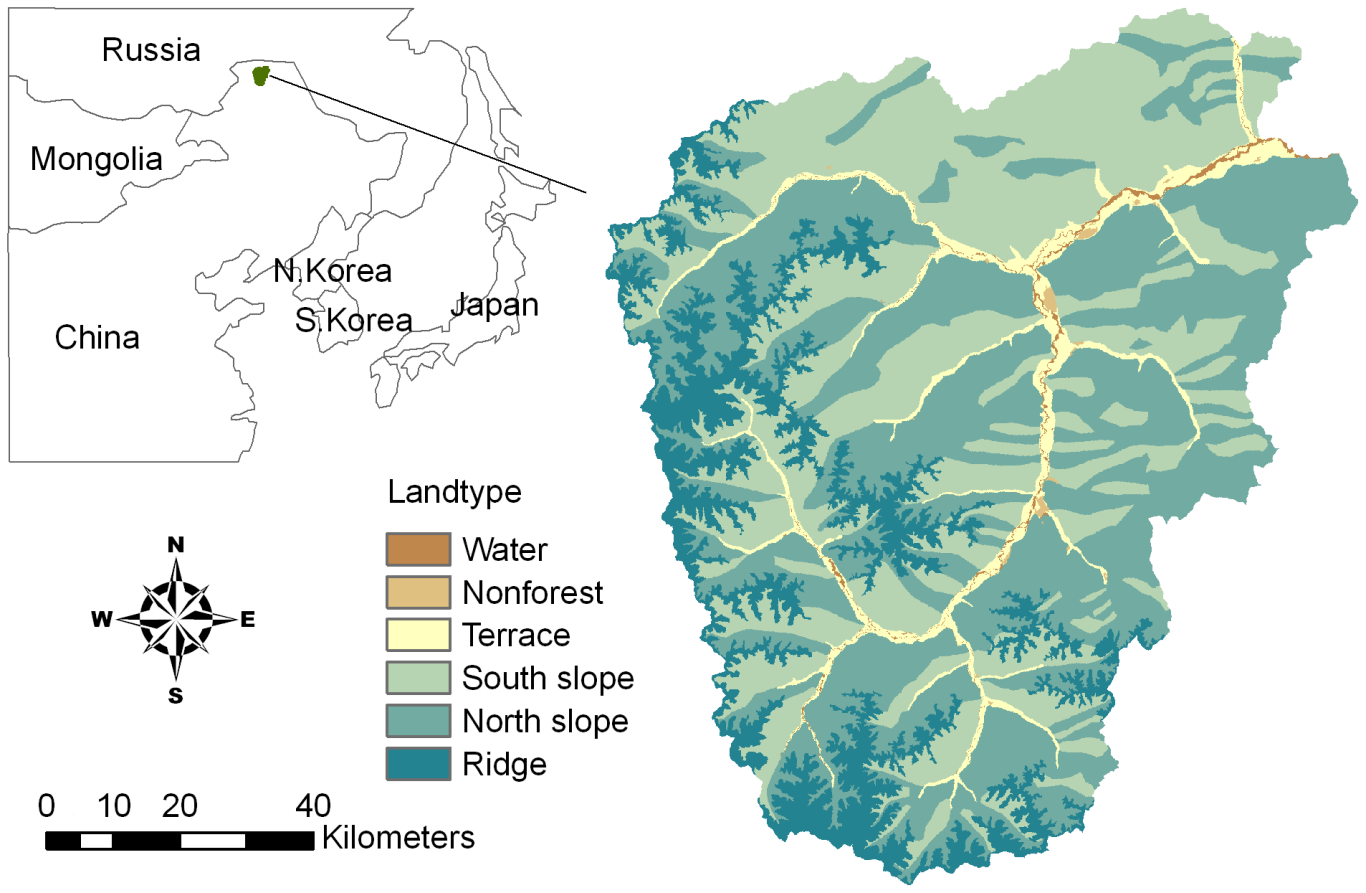
The geographic location of the study area and different land types, among which water and nonforest are not simulated in the model.

Vegetation in this region falls within the cool temperate coniferous forests occurring at the southern extension of the eastern Siberian light coniferous forest [36]. Canopy trees include larch (*Larix gmelini*), Mongolian Scots pine (*Pinus sylvestris* var. *mongolica*), Korean spruce (*Picea koraiensis*), birch (*Betula platyphylla*), aspen (*Populus davidiana*), poplar (*Populus suaveolens*), willow (*Chosenia arbutifolia*), and a shrub species *Pinus pumila* (dwarf pine) (Table 1). Larch, comprises about 78.4% of the forest area, and is the dominant and most widely distributed tree species except in some riparian wetlands. Birch, a pioneer species with strong colonization ability, always coexists with larch after burning and harvesting. Mongolian Scots pine, with its limited spatial distribution, is always mixed with larch. Dwarf pine occurs with larch or birch where t elevation is over 700 m. Willow and poplar are confined to along the riverbanks. Because larch and birch are the two most widely distributed species in this region, for convenience we refer to these as major species and the others as minor species to notate conveniently. Both the species composition of vegetation in Huzhong Forestry Bureau and in Huzhong Natural Reserve is similar. Although forests both in the forestry bureau and the natural reserve have succeeded into old growth stage (200–300 years), forests in the forestry bureau due to timber harvesting mostly are early stage (40–80 years). We divided the area not covered by forest into water and non-forest areas which collectively often serve as fire breaks. We also divide the forest area into four topographic subdivisions (land types: terrace, south slope, north slope, and ridge) to better consider the effects of microclimate, soil, and complex topography on species distributions (Figure 1).

**Table 1:** Species life-history attributes for canopy species in northeastern China.

Species	Common name	LONG	MTR	SHD	FIRE	EFFD	MAXD	VGP	MVP	CSEPs	WSEPs	DMAX	DMIN
*Betula platyphylla*	Birch	120	15	1	3	0	2000	1	20	0.153	0.341	3100	600
*Populus davidiana*	Aspen	100	10	1	1	0	2000	1	15	0.010	0.178	3000	800
*Populus suaveolens*	Poplar	180	12	1	2	0	2000	1	15	0.013	0.048	1900	400
*Chosenia arbutifolia*	Willow	250	18	2	1	0	3000	0.9	15	0.018	0.054	2400	600
*Larix gmelinii*	Larch	300	20	2	5	50	150	0	0	0.288	0.136	1900	400
*Picea koraiensis*	Spruce	300	30	4	1	100	150	0	0	0.060	0.112	2500	800
*Pinus sylvestris* var. *mongolica*	Mongolian Scots pine	250	40	2	4	100	200	0	0	0.175	0.212	2400	700
*Pinus pumila*	Dwarf pine	250	30	3	3	50	100	0	0	0.225	0.144	1400	300

LONG, longevity of the species (years); MTR, maturity age of the species (years); SHD, shade tolerance value (1–5) (no units), 1 = least tolerant, 5 = most tolerant; FIRE, fire tolerance value (1–5) (no nits), 1 = least tolerant, 5 = most tolerant; EFFD, species effective distance seeding range (m); MAXD, species maximum distance seeding range (m); VGP, probability of vegetative propagation following disturbance (no units); MVP, minimum age of vegetative propagation (years); CSEPs, probability of species establishment under current climate; WSEPs, probability of species establishment under climate warming scenario; DMAX, maximum growing degree day; DMIN, minimum growing degree day each species.

Wildfire was the major disturbance in the Great Xing’an mountains before the forest was exploited by humans, and it is one of the more important factors that drive forest succession [37], [38]. Based on Chinese Federal Forest Service data, lightning-caused fires burning 131603.6 ha and 14% of the landscape account for about 94% of the total fires and nearly 99% of the total burned area during the 40-year period from 1965 to 2005 for our study area. The forest in this region has been exploited by humans in various ways since the 1950s, and timber harvesting has significantly impacted forest age structure and natural regeneration [37]. Based on Chinese Federal Forest Service data, larch forests have shifted from late-seral or old-growth stages to a mid-seral stage. Consequently, to maintain older age classes and therefore forest integrity and sustainability, timber harvesting has been restricted by the government since 1999. Hence, the age structure of forests in this region has been shaped primarily by burning and timber harvesting. Mature larch stands, which develop after multiple surface fires have killed ground vegetation and shrubs, allow new cohorts of tree reproduction to establish after each fire, which then form unevenaged stands (≥3 age classes). All other stands established after stand-replacing fire or harvest are evenaged.

### 2. Design of Simulation Experiments

To assess the effects of climate warming, increased frequency of burning, and timber harvesting on forest landscapes under future climate scenarios, we designed a factorial experiment with three independent variables each with two levels: climate (current vs. expected future), fire occurrence (current vs. increased fire), and timber harvesting (no harvest vs. legal harvest). Each treatment combination was simulated at 10-year steps over 300 years (1990–2290) replicated with five times which meets the minimum number for the statistic requirement.

#### 2.1 Climate warming scenarios

Current climate data were obtained from the Northeastern Regional Meteorological Center in China, and daily temperatures and precipitations were compiled for 1961–1990. Average monthly temperature and precipitation were derived from daily temperature and precipitation data. To process climate data for the current and warming scenarios we first used data from six weather stations distributed across the northern Great Xing’an mountains to develop monthly temperature and precipitation values along the longitudes, latitudes and elevations of the mountains. We then calculated monthly temperatures and precipitations based on the developed relationships [39].

Expected future climate parameters were estimated based on projections of the Hadley GCM (UKMO-HadCM3) running under the A2 scenario for 2070–2099. The A2 scenario represents high CO_2_ emissions (1250 ppm) related to high human population size and slow technological adaptation (IPCC 2007). We chose the Hadley GCM because it is widely accepted and provides the method currently considered the best for detecting climate warming effects. It predicts warmer and moister summers compared to many other GCMs. We obtained changes (projected by the Hadley GCM) in monthly average temperature and precipitation over the next 100 years from ClimateWizard [40], and recorded the data as a gridded dataset with a 0.5×0.5° resolution. The Hadley GCM projected that annual average temperature and annual precipitation would increase by 5°C and ∼35%, respectively by the 2080s. We modified current climate means and monthly weather records by adding the change in temperature (°C) and precipitation (mm) between the Hadley projection for the periods 1961–1990 and 2070–2099. During the 21st century, we assumed that precipitation and temperature trends are linear and that variability in temperature and precipitation would not change [41].

#### 2.2 Fire regimes under two climate scenarios

The historical fire regimes (including fire ignition probability, mean return interval, mean fire size, and standard deviation of fire size) for our simulations were parameterized from a fire database from 1990 to 2000. According to Chinese Federal Forest Service data (1990–2000), mean return interval is 238 year, mean fire size is 1884 ha, and fire ignition probability is 0.00402 for the historical fire regimes. Under the A2 scenario, both the fire ignition probability and mean fire size was increased by 200% based on historical regimes, and mean return interval was decreased to 1/3 of the historical regimes. Liu et al. [28] predicted that fire occurrence of boreal forest in the northeast China under the A2 climate change scenario projected by the Hadley GCM would increase by 230% in 2100.

#### 2.3 Forest management scenarios

Our study area is divided into three forest management sub-areas: areas where cutting is banned (50.1%), areas with restricted cutting (23.08%), and areas where timber harvesting is permitted (26.82%). Each of these sub-areas is further divided into compartments (stands) with an average size of 20 ha. Harvest units do not exceed 10 ha in size, and adjacent stands are not harvested for at least five years. Current forest managements are different in specific sub-areas for the study area (Table 2). In our study, we assumed that only legal harvesting occurred and that harvesting regimes did not vary. To select stands for harvest, we applied the oldest-first method, in which all stands within a management area were ranked by age and stands of oldest age were harvested first. We also set the minimum stand age for harvest at 40 years, reflecting the current harvest practices. All species except dwarf pine and willow were harvested. Dwarf pine is not harvested because it is a key shrub species for maintaining habitats ≥1000 m elevation; similarly willow is crucial to maintaining riparian habits.

**Table 2:** Parameters of harvest scenario.

Species	Age range (year)		% Area harvested (of each management area per decade		
		Cutting method	Harvest area	Restricted cutting area	Regeneration
Larch	120–300	clearcut	0.5%	0.3%	natural
Mongolian Scots Pine	90–250	clearcut	0.5%	0.3%	natural
Spruce	120–300	clearcut	0.5%	0.3%	natural
Birch	60–150	clearcut	0.5%	0.3%	natural
Aspen	40–120	clearcut	0.5%	0.3%	natural
Poplar	50–180	clearcut	0.5%	0.3%	natural

The harvest scenario was adopted from current forest management of Huzhong Forest Bureau and was parameterized in LANDIS harvest module.

### 3. Model Simulation

We used a forest landscape model (LANDIS) to simulate forest landscape dynamics under different climate, fire, and harvesting scenarios. LANDIS can be used to simulate forest landscape changes related to forest succession and disturbances at large heterogeneous spatial extents (10^3^–10^7^ ha) over long time spans (10–1000 years) based on raster data in which each cell contains unique information relating to specific species, age cohorts, and time since last disturbance.

We used LANDIS modules (forest succession and seed dispersal, fire disturbances, and harvesting) to simulate forest landscape changes of the study area under different climate scenarios. LANDIS simplifies within-stand processes and individual tree information, and tracks the presence or absence of species age cohorts to simulate succession. Succession at each stand is a competitive process driven by species life history attributes such as longevity, age of sexual maturity, shade tolerance class, fire tolerance class, the minimum age of vegetation sprouting, sprouting probability, and effective and maximum seeding distance. Succession at the landscape scale involves seed spatial dispersal among cells and the different capability for species establishment on each land type [34], [42].

LANDIS simulates seed dispersal using an exponential distribution in which the effective and maximum dispersal distances of specific species control seedling distribution [42]. When seeds successfully arrive at a site, the shade-tolerance rank of the seedling relative to the species existing on the site determines the recruitment of the seedling. Whether the seedling successfully establishes and survives is determined by the specie establishment probability (SEP, a value ranging from 0–1). The species establishment probability quantifies how a species establishes in different environmental conditions. Species with high establishment probability have higher probabilities of establishment, and are as responses of tree species to climate in LANDIS. SEPs as input to LANDIS are estimated based on existing experimental data [43] or derived from the simulation results of a gap model [35] such as LINKAGES.

In our study, we employed LINKAGES (a derivative of the JABOWA/FORET class of gap models) [44] to simulate the physiological response of tree species to both current and warming climate within each land type. The physiological response was quantified as individual species biomass, and was used to estimate the SEP for specific species. Individual species biomass was determined by simulating the interactions of climate, soil properties (derived from soil survey data in the study area), and species biological traits (compiled based on previous studies in this area) with ecological processes. The climate properties utilized included monthly temperature and precipitation (Table S1 in Appendix S1). The soil properties included field water capacity, wilting point, total nitrogen, and total carbon. The species biological traits included longevity, maturity, shade and drought tolerance, and seedling capability [36], [37]. The ecological processes simulated were competition, succession, and water and nutrient cycling.

To examine variations in species establishment by land type we simulated one species at a time in LINKAGES planting the same number of trees (200 saplings/ha) for each land type [35]. We converted the simulated biomass for all land types under both current and warming climate to two sets of SEPs (Table S2 in Appendix S1) using an empirical method [35] (S1 in Appendix S1).

To simulate a gradual change in climate under the currently expected scenario, SEPs for the climate treatment initially assumed current values and then were modified by a 10-year-interval linear interpolation of values calculated from simulated year 0 to year 100. After 100 years, model values were held constant. Our estimates are probably conservative, because warmer conditions are expected to become more pronounced after 2090 [7].

In LANDIS, fire is simulated as a stochastic process based on the ignition probability distribution, mean return interval and mean size characterized for various land types [34], [45]. The fire module simulates temporal patterns of fire regimes using a hierarchical fire frequency model, which divides a fire occurrence into two consecutive events: fire ignition and fire initiation. Fire ignition is generated from the Poisson distribution based on the fire ignition density defined in fire regimes. Whether a fire ignition can result in fire initiation is dependent on the fuel loading, fuel arrangement, and fuel moisture content. A fire initiation event starts with the ignition and is successful when an area equal to the cell size is burned. For each fire initiation, LANDIS simulates fire spread using a modified percolation method spread from a burning cell to forested cells in the cardinal directions (north, northeast, east, southeast, south, southwest, west, northwest). These cells are randomly entered into a priority queue. The fire will spread by randomly selecting a fire size using a log-normal distribution based on mean fire size and standard deviation of fire size. Fire intensity is determined by the time since the last fire on the site, as well as the amount of fuel present within each cell. In LANDIS, small or young trees are more susceptible to fire than large or older trees. LANDIS simulates fire intensity from low intensity ground fires to high intensity crown fires as five levels. Correspondingly, trees are sorted into five-fire tolerance classes. Fire severity is the interaction of species fire tolerance, species age cohorts, and fire intensity [34].

In the harvest module of LANDIS, timber harvests are simulated within a specific hierarchical management structure. The overall landscape is divided into forest management areas, each to be treated by specific harvest regimes at specific intensities. Each management area is divided into stands of various forest types. Each stand includes a group of grid cells being populated with a specific combination of species and age cohorts. Within each management area, harvests are implemented by removing specific cohorts of specific species on sites selected for harvest based on harvest regimes. The harvest regimes prescribe the harvest rules (e.g., how to harvest a stand, how to allocate a harvest based on stand ranking which in turn is based on ecological or economic criteria, and how to harvest age cohorts of tree species such as shelterwood, selection, and clear cutting) [33]. The harvest regime is controlled by users based on the targets of managers, and it is determined by the combination of temporal, spatial, and species composition [33].

Various components and processes have been described elsewhere [32]–[34], [42]. The effectiveness of the LANDIS model in boreal forest ecosystems in northeastern China has been demonstrated in previous studies [46]–[48]. The uncertainty analysis on model parameterization and result variations has been previously done by Xu et al. [48], [49]. Their research showed that the uncertainty was low at the beginning of the simulation, increased with simulation year, and finally reached an equilibrium state, where the uncertainties of input parameters had little effect on the simulation results (species percent area and their spatial patterns) at the landscape level. To simulate landscape processes, LANDIS requires setting variable parameters and creating maps for model initialization. Maps delineate forest composition, land types, and type of forest management permitted. Parameters include species vital attributes (Table 1), species establishment probabilities (SEPs), harvest regime attributes, and fire regime attributes. The data for parameterization of LANDIS include the forestry inventory taken in 1990 in the study area, two Landsat TM scenes taken in 1990, fire records from 1990 to 2000, and a Digital Elevation Model (DEM) generated from the contour lines delineated in 1971. These parameters and maps have been specified in previous studies [48], [50].

### 4. Data Analysis

Landscape responses to climate warming, burning, and timber harvesting were quantified in related to species composition, tree age structure, and forest landscape pattern. Model outputs primarily were derived from maps showing the effects of the three independent variables on species composition. Maps were produced for simulations at each 10-year time step. We chose a 10-year time step for mapping the dominant species and the maximum cohort age of all species combined. Species composition was expressed as the proportion of the landscape dominated by each species. We examined forest age structure using five age-based stages: 1–40 years (establishment stage), 41–100 years (early-stage), 101–140 years (mid- stage), 141–180 years (late-stage), and >180 years (old-growth stage) (Gustafson et al., 2010). To quantify spatial pattern of seral stage and the major species (larch and birch) we used an aggregation index (AI) which reflects the tendency of like cells to be adjacent [51], and Shannon diversity index (SHDI) which reflects the heterogeneity of a landscape [52].

The effects of climate warming, forest burning, and timber harvesting were analyzed using multiple analyses of variance (MANOVA). With this method, we tested (for the significance of each independent variable) effect on the dependent variable. We ran separate analyses for each response variable (species composition, tree age structure, and spatial pattern). We chose a subset of representative variables to reduce multi-collinearity within each analysis (Table 3). Because the response variables varied through time, we chose simulation-years 150 and 300 (actual years 2140 and 2290) as representative of the varying response (Table 3). MANOVA models used the error sums of squares and cross products (residual) matrix, and the results were evaluated using Type I sums of squares. The relative effects of every treatment were quantified as the percent of the total variation attributed to each effects and significance was judged conservatively using at α = 0.01. Our explanations focused on trends rather than statistical significance because random noise in the tightly controlled simulations was minimal.

**Table 3:** Comparison of forest composition outside the natural reserve under the current climate to observed value in Huzhong natural reserve.

	Huzhong natural reserve	Outside the natural reserve		
	%Observed	%Initial conditions	% Range (years 200–300)	%Mean (years 200–300)
Species composition				
Aspen	1.1	4.5	2.0–2.1	2.1
Birch	33.1	34.1	29.0–33.3	32.4
Poplar	1.1	0.5	0.2–0.6	0.4
Willow	1.2	0.7	1.1–1.3	1.2
Larch	52.9	44.7	45.9–51.0	47.3
Spruce	1.0	1.3	2.6–3.4	3.0
Mongolian Scots pine	1.4	5.0	3.3–3.5	3.4
Dwarf pine	7.2	9.3	10.2–10.4	10.4
Age composition				
Establishment (1–40 yr)	3.4	12.9	3.7–6.0	4.5
Early-stage (41–100 yr)	9.6	42.8	8.8–13.0	10.4
Mid-stage (101–140 yr)	10.5	32.5	10.9–14.6	11.9
Late-stage (141–180 yr)	20.2	11.5	24.7–29.8	19.1
Old-growth (>180 yr)	56.3	0.3	47.8–62.5	54.2

### 5. Simulation Result Verification

Simulation result validation requires long term spatial and temporal data, which are not available. This is especially true for the climate warming scenario. To gain assurance of the simulation results we compared species composition and age composition of simulation years 200–300 to those from the natural reserve in our study area. Forests in the natural reserve have reached the old growth stage with ages ranging from 200 to 300 years. Thus, results from simulation year 200–300 provide legitimate comparisons for result verification.

## Results

### 1. Results Verification

Simulation of the current climate and disturbance regimes (fire and harvesting) showed that the mean of the proportions for most species and age classes were similar to the observed from Huzhong Natural Reserve (Table 3). The ranges of simulated proportions for most species and age classed were coincident. Only the ranges of simulated the proportions for larch and birch were discrepant.

### 2. Species Composition Responses to Climate and Fire Under the Current Harvest Regime

**Figure 2:**
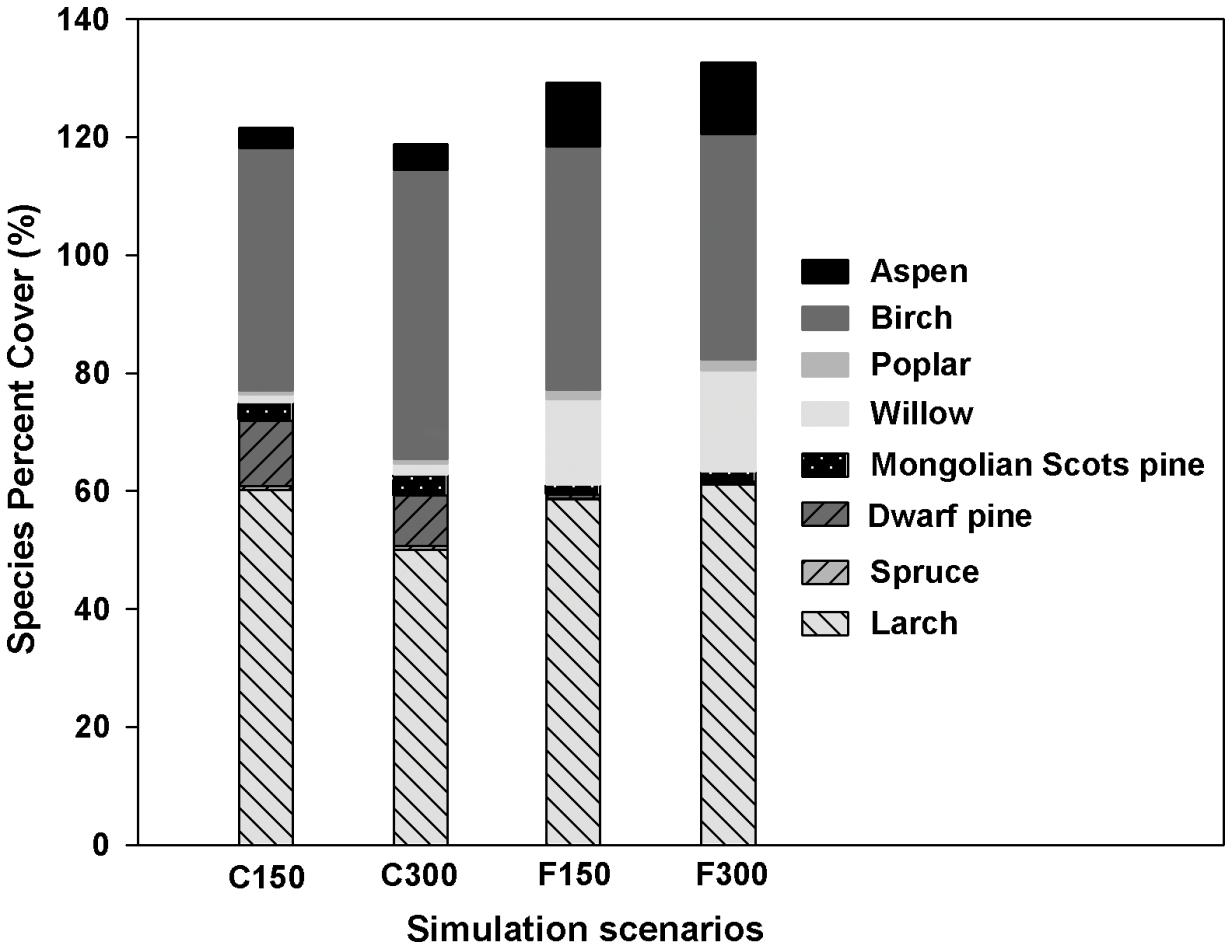
Responses of species composition to climate and fire under current harvest regime. C150 represents the average of the simulated species abundance under current climate and fire regimes in 2000–2140 years; C300 represents the average of the simulated species abundance under current climate and fire regimes in 2150–2290 years; F150 represents the average of the simulated species abundance under future climate and fire regimes in 2000–2140 years; and F300 represents the average of the simulated species abundance under future climate and fire regimes in 2150–2290 years.

The abundance of willow, poplar, and aspen for a given future climate and fire regime can be expected to significantly increase compared to the abundance of these species under current climate and fire regimes. Moreover, the simulations showed that the abundance of conifers such as spruce and the two pines greatly decreased whereas birch abundance decreased more slowly. The simulations also indicated that the predominant tree species in this region may be expected to change from predominantly conifers to broadleaf species (Figure 2).

### 3. Effects of Climate Warming, Burning, and Harvesting on Forest Species Composition

The simulations show that tree species composition is be expected to be strongly impacted by climate warming, fire, and harvest (Table 4). Simulated climate warming significantly increased the abundance of most species, and its effects on most species were greater in year 2290 than in year 2140. The effects of climate warming on the abundance of aspen, poplar, and willow were major compared to the main effects of fire and timber harvest. However, effects of climate warming on the abundance of birch, larch, spruce, and pines were much smaller than effects of fire and timber harvest. Burning mostly reduced the abundance of spruce and pines in both year 2140 and year 2290, and its effects were greater in year 2140 than in year 2290. Increased fire strongly increased larch abundance and decreased birch abundance in year 2290. Birch and larch, the major species in this region were mostly impacted by harvest. The harvest treatment increased birch abundance and decreased larch abundance in both years, and its effects were markedly smaller in year 2290 than in year 2140.

**Table 4:** MANOVA results for species composition variables.

		Climate effect 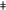		Fire effect£		Harvest effect§		Fire×harvest	
Simulation years	Species (%)	Variation explained (%)	t	Variation explained (%)	t	Variation explained (%)	t	Variation explained (%)	R^2^
150 (2140)	Aspen	79.8**	**26.3**	3.0**	**4.5**	13.0**	**8.4**	0.2	0.96
	Birch	0.3	**4.9**	1.3**	**17.9**	96.0**	**77.7**	2.0**	1.00
	Poplar	61.3**	**12.7**	12.1**	**5.1**	12.5**	**5.1**	0.9	0.85
	Willow	90.3**	**26**	0.06	0.63	4.6**	**5.3**	0.3	0.95
	Larch	5.3**	**16.5**	24.8**	**−30.2**	68.1**	**−46.7**	1.0**	0.99
	Spruce	5.8**	**6.9**	88.4**	**−20.6**	0.9**	**−3.6**	0.6	0.95
	Mongolian Scots pine	0.3	1.4	78.8**	**−17**	1.8**	0.34	1.3**	0.94
	Dwarf pine	0.02	**−3.7**	99.2**	**−159.1**	0.4	**19.5**	0.3	1.00
300 (2290)	Aspen	92.9**	**49.5**	2.1**	**−5.4**	3.7**	**6.9**	0.001	0.99
	Birch	1.7**	**−7.7**	58.6**	**−30.8**	38.6**	**25.9**	0.02	0.99
	Poplar	82.4**	**13.3**	0.3	**−**0.64	1.0**	0.91	0.01**	0.82
	Willow	81.0**	**17.5**	7.9**	**−3.3**	1.7**	2.3	0.1	0.90
	Larch	0.7**	**3.7**	37.1**	**15**	58.4**	**−29.5**	2.2**	0.98
	Spruce	10.9**	**6.2**	78.4**	**−12.7**	0.6	**−**1.9	0.4	0.89
	Mongolian Scots pine	39.4**	**13**	51.7**	**−17.8**	11.0**	**3.8**	0.3	0.89
	Dwarf pine	0.01	**−**1.6	97.7**	**−118.8**	1.3**	**28.2**	1.0**	1.00

The t values test the hypothesis that the response between levels of main effects are equal, and significant (α = 0.01) differences are indicated in boldface. All three main effects were significant in both years. Only the fire × harvest interaction was always significant and was included in the model. Significant interactions are indicated by asterisks.

** P<0.01.

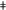
Positive t value means that response variable increases as climate warms.

£ Positive t value means that response variable increases as fires increases.

§ Positive t value means that response variable increases when harvest is added.

### 4. Effects of Climate Warming, Burning, and Harvesting on Forest Age Structure

Forest age structure was impacted more strongly by fire and harvest rather than climate warming, although direct climate warming effects would increase the SEPs of most species (Table 5). Increased fire had positive effects on the abundance of tree establishment in both year 2140 and year 2290 and had strong negative effects on their abundance during mid-stage and late-stage in both years, and these effects were greater in year 2140 than in year 2290. Harvesting strongly increased tree abundance in the early stage and decreased its abundance in the late stage and old-growth stage, and these effects were markedly greater in year 2290 than in year 2140.

**Table 5:** MANOVA results for forest age structure variables.

		Climate effect 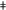		Fire effect£		Harvest effect§		Fire×harvest	
Simulation years	Seral stage (%)	Variation explained (%)	t	Variation explained (%)	t	Variation explained (%)	t	Variation explained (%)	R^2^
150 (2140)	Establishment	0.4**	**12.2**	40.8**	**77.9**	58.6**	**94.1**	0.1**	1.00
	Early-stage	7.4**	**15.2**	5.3**	**12.2**	85.5**	**39.6**	0.6**	0.99
	Mid-stage	0.7**	**22.1**	97.3**	**−155.9**	0.08	**19.7**	1.8**	1.00
	Late-stage	0.002	1.5	77.0**	**−239.6**	22.2**	**−116.4**	0.8**	1.00
	Old-growth	0	0.77	5.1**	**−106.2**	94.8**	**−419**	0.09**	1.00
300 (2290)	Establishment	0.2**	**−4.3**	8.6**	**12**	89.4**	**57**	1.4**	1.00
	Early-stage	2.0**	**10.9**	6.6**	**−14.1**	90.8**	**51.6**	0	0.99
	Mid-stage	28.2**	**9.6**	52.2**	**−7.6**	7.3**	**−**1.9	1.5	0.88
	Late-stage	5.6**	**14.9**	15.5**	**−6.5**	71.9**	**−26.7**	6.1**	0.99
	Old-growth	0.2**	**16.4**	0.09**	**8.4**	99.2**	**−220.3**	0.4**	1.00

The t values test the hypothesis that the response between levels of main effects are equal, and significant (α = 0.01) differences are indicated in boldface. All three main effects were significant in both years. Only the fire × harvest interaction was always significant and was included in the model. Significant interactions are indicated by asterisks.

** P<0.01.

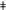
Positive t value means that response variable increases as climate warms.

£ Positive t value means that response variable increases as fires increases.

§ Positive t value means that response variable increases when harvest is added.

### 5. Effects of Climate Warming, Burning, and Harvesting on Forest Landscape Pattern

The spatial pattern of the aggregation index (AI) and Shannon’s diversity index (SHDI) responded strongly to increased fire and harvest (Table 6). Climate warming significantly affected forest fragmentation and forest diversity. However, its effects were markedly smaller than the effects of increased fire and harvesting. Both increased fire and timber harvesting would increase forest fragmentation and decrease forest diversity. However, increased fire was the predominant influence in both the fragmentation and the diversity of age classes; an exception was the diversity of age classes in year 2290. Harvesting impacts were greater for the pattern-of-response variables for birch and larch. The arithmetic sign and relative strength of these effects were not always consistent through time (e.g., AI-larch and SHDI-birch for timber harvesting effects Table 6). For example, larch was the major tree species that is harvested in this region and was also the dominant species in the early years of simulations. Harvest effects on the aggregation index of larch (AI-larch) in year 2140 were significant (Table 6). However, the abundance of larch rapidly decreased through time due to the action of various disturbances and succession. Thus, harvesting effect on AI-larch was greatly reduced by year 2290.

**Table 6:** MANOVA results for spatial pattern variables.

		Climate effect 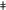		Fire effect £		Harvest effect§		Fire×harvest	
Simulation years	Pattern index	Variation explained (%)	T	Variation explained (%)	T	Variation explained (%)	t	Variation explained (%)	R^2^
150 (2140)	AI-seral stage	2.4**	**14**	52.3**	**−40.8**	44.2**	**−37.1**	0.7**	1.00
	AI-birch	0.2	**−10**	0.2	**20.2**	95.9**	**−113.4**	3.5**	1.00
	AI-larch	4.6**	**10**	26.3**	**−15.5**	67.3**	**−25.7**	0.2	0.98
	SHDI-seral stage	0.9**	**20**	52.4**	**−70.1**	40.3**	**−56.9**	6.3**	1.00
	SHDI-birch	0.5**	**6.6**	4.8	**28.2**	90.4**	**77.8**	3.9**	1.00
	SHDI-larch	2.3**	**−18.7**	45.9**	**80.3**	45.9**	**80.3**	5.8**	1.00
300 (2290)	AI-seral stage	8.0**	**41.9**	83.1**	**−89.8**	8.4**	**−24.9**	0.3**	1.00
	AI-birch	1.5**	**−10**	0.03	**21.1**	83.5**	**−31**	14.5**	0.99
	AI-larch	33.5**	**8.5**	5.4**	1	34.0**	**−**2.6	11.0**	0.82
	SHDI-seral stage	8.9**	**13.2**	1.9**	2.6	82.6**	**−21.5**	4.8**	0.98
	SHDI-birch	0.007	-0.27	19.8**	**−11.3**	76.9**	**19.7**	0.14**	0.96
	SHDI-larch	7.4**	**−14.7**	23.5**	**17.6**	67.9**	**30.6**	0.07	0.99

The t values test the hypothesis that the response between levels of main effects are equal, and significant (α = 0.01) differences are indicated in boldface. All three main effects were significant in both years. Only the fire × harvest interaction was always significant and was included in the model. Significant interactions are indicated by asterisks.

** P<0.01.

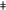
Positive t value means that response variable increases as climate warms.

£ Positive t value means that response variable increases as fires increases.

§ Positive t value means that response variable increases when harvest is added.

AI is the aggregation index of He et al. (2000) that reflects the tendency of like cells to be adjacent, SHDI is Shannon diversity index that reflects the heterogeneity of landscape.

## Discussion

We estimated the relative effects of climate warming, burning, and timber harvesting on forest landscapes in northeastern China. The results showed that every treatment (climate, fire, and timber harvest) had strong effects on forest composition and forest spatial pattern. The effects of climate warming on tree species composition were significant but had a lag time (Table 4). However, forest age structure was mostly impacted by forest disturbance rather than direct climate changes (Table 5). This effect is likely related to the direct effects of climate on the abundance birch, larch, spruce, and two pines, which were smaller than the effects of disturbance. This is consistent with previous studies that have shown that direct effects of climate warming on forest composition are not as great as effects of harvesting and increased fire [29]–[31].

**Figure 3:**
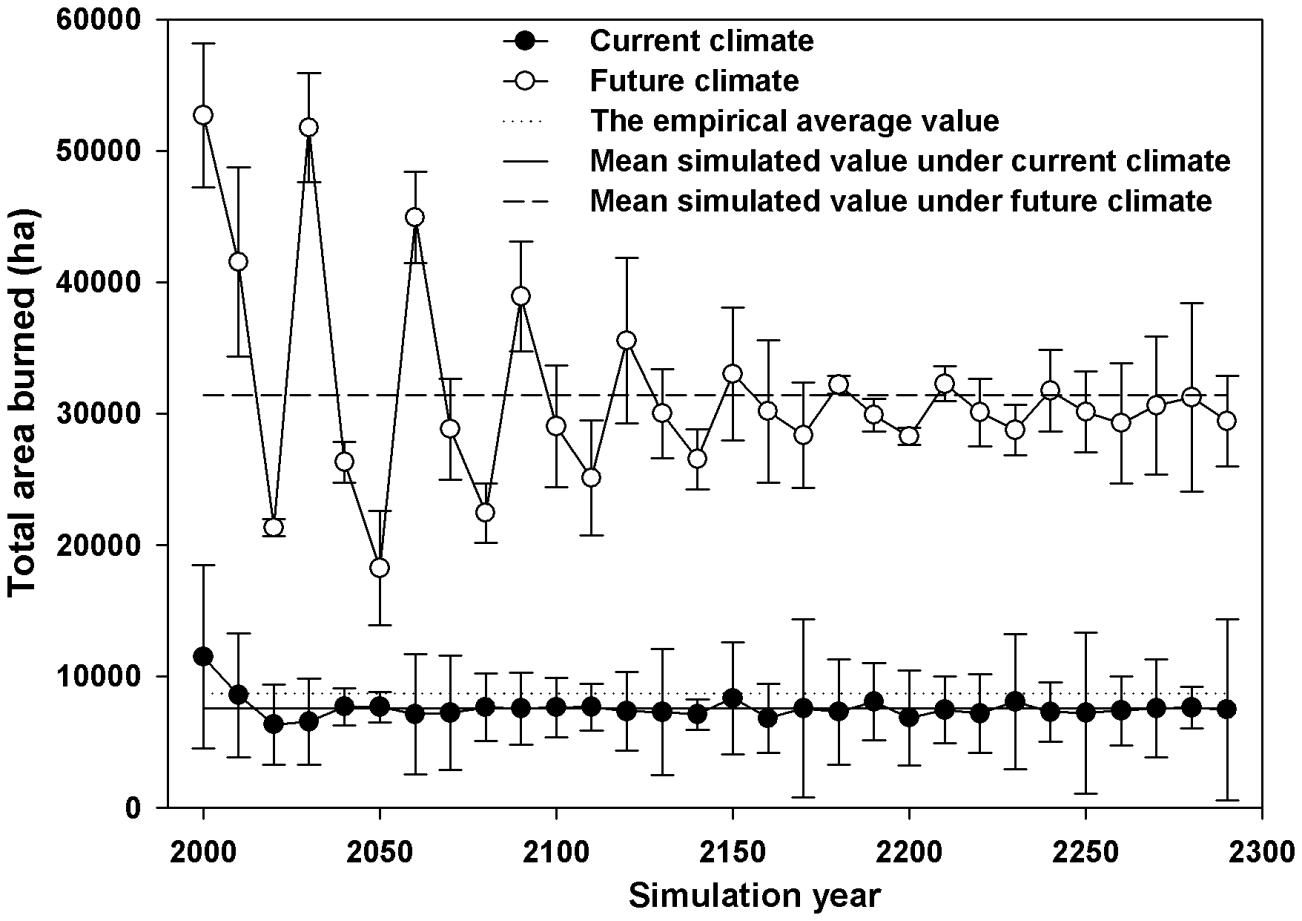
Total area burned per decade and the size distribution of fires (±SD) for 300 simulation years under current climate and future climate.

In our study, fire regime was specified primarily by area burned, fire size, and fire severity (i.e., underground fire, surface fires, and canopy fires). We reproduced the empirical (current) fire regime quite closely, and found that the total area burned fluctuated in a small range after the year 200 simulation when climate warming continued to exacerbate conditions (Figure 3). As expected, fire had strong effects on forest composition. Fire overshadowed the direct effects of climate warming on the abundance of spruce, pines, and larch. Moreover, fire effects on broadleaf species were much smaller than on coniferous species. This is consistent with the results of He et al. [32], which also showed that increased fire can accelerate the decline of shade-tolerant species. However, potential effects of increased fire may be variable because fire events are highly variable in both size and frequency [23], [27], [28].

Timber harvesting nevertheless can be expected to strongly impact forest composition in regions undergoing climatic change. In our study region, this is largely the result of changes in the abundance of the major species (birch and larch) and the abundance of late-stage and old-growth tree age classes. This is consistent with the results of Gustafson et al. [31] and He et al. [32]. Gustafson et al. [31] showed that harvest effects were more significant than the effects of climate warming in south-central Siberia. The forests in the Great Xing’an mountains have long been exploited by human, and the resulting effects have thus been continuous and complex. However, timber harvesting and other human activities can be potentially controlled by managers. Therefore, we propose that our modeling approach should be useful in evaluating alternative management policies to mitigate at least some of the negative effects associated with climate warming.

In our study, climate warming was significantly and directly related to forest landscape pattern (Table 6). Nevertheless, those effects were overshadowed by fire or harvesting. Similar to the results of Gustafson et al. [31], timber harvesting in our study also increased forest fragmentation. Increased fire altered the direct effect of climate change on forest fragmentation and the diversity of the pattern of tree age classes. Timber harvesting also altered the fragmentation and diversity of the compositional pattern of tree species. In our study, we selected only the aggregation index (AI) and Shannon’s diversity index (SHDI) to represent fragmentation and diversity. It would require more detailed analysis of changes on the landscape including tree species composition, patch size, and connectivity relative to specific species’ life requirements to arrive at more specific conclusions.

Our results nevertheless suggest that changes in forest landscapes are complex and involve continuous interactions among climate, fire, and timber harvesting effects. The modeling approach we used can be used to evaluate forest management policy options for mitigating negative effects of climate warming. When interpreting our simulation results, it is important to note the following limitations:

1. We only incorporated the effects of alteration of temperature and precipitation on forests; however, tree growth is impacted by changes in solar irradiation and CO_2_ fertilization [53].
2. Climate warming indirectly influences forest landscapes through tree species migration, shifts in soil nitrogen deposition [54], changes in natural disturbance regimes such as fires [20] and insect outbreaks [55]. However, we only incorporated changes in the fire regimes.
3. Fire events are highly variable in size and frequency [24], [27]. Krawchuk et al. [24] illustrated that area burned would increase 1.9-fold by 2040–2049 and 2.6-fold by 2080–2089 relative to 1975–1985 conditions. However, we initially parameterized future fire regimes by increasing by 2-fold of the current fire occurrence based on Liu et al. [28].
4. Spatial pattern dynamics are related to the resolution and grid cell structure used to represent the landscape. The grain size of the initial conditions map may influence the effects of disturbances, because if the grain of a disturbance is the same as the existing pattern, changes in pattern metrics are less likely to be detected.

These limitations provide cautions on concluding that our model makes robust predictions of the future forest dynamics. However, our results should be as reliable as those presented in similar studies.

LANDIS is primarily a process-based model. Its parameters and algorithms are generally accepted as adequate representation of forest dynamics, and that the treatments imposed by modifying parameters related to climate and disturbances should provide useful insight into landscape change. Like other forest landscape models, LANDIS conceptually simulates non-spatial processes and assumes how they interact with landscape processes and with each other. For many processes their behaviors are clearly understood and assumptions are firmly established, but for others less is known about the true behavior of processes. For example, LANDIS simplifies individual tree information and within-stand process and only tracks the presence and absence of species age cohorts for simulating forest succession [42]. The model nevertheless allows large scale questions such as spatial pattern, species distribution, and disturbances to be effectively addressed. When it simulates fires, it performs a Bernoulli trial to address fire ignition, and then randomly select a fire size from a log-normal distribution which is recognized as a distribution useful in simulating fire spread. But fire ignition and spread factually depend upon the combination of weather, fuels, and topography. LANDIS also implements timber harvests within a specific hierarchical management structure, and it removes specific cohorts by specific species on sites selected for timber harvest which are defined by harvest regimes [33]. However, timber harvest and fire also impacts the growth of tree species by affecting the availability of soil nutrients which is a factor not included in LANDIS [56]. Nevertheless our results were reliable, one of the reasons is that LANDIS simulations have been widely used in many simulation studies and its validity reported in other studies in Northeast China [46]–[48]. Using LANDIS to conduct a controlled simulation experiment allows discovery of general trends in boreal forest responses to climate warming, burning, and harvesting based on our current understanding of the ecological processes that drive forest dynamics.

### Conclusions

From our study, we concluded that: (1) the composition of forests of the Great Xing’an mountains is likely to be significantly altered by changing climate, timber harvesting, and burning. (2) The direct effects of climatic change in the study area are not likely to be as important as timber harvesting and the potential for increased burning. (3) Disturbance by burning and harvesting may greatly reduce the abundance of conifer species including larch, spruce, and two pine species. In turn, this may significantly reduce the ecological integrity of these forests by decreasing tree species diversity and increasing forest fragmentation.

## Supporting Information

Appendix S1 **Estimation of species establishment probability**. Table S1 The monthly temperature and precipitation under current climate and future climate. Table S2 Species establishment probability of specific species under two climate scenarios.
(DOC)
