# Supplementary material for: Comparing Effects of Climate Warming, Fire, and Timber Harvesting on a Boreal Forest Landscape in Northeastern China
Source: PLoS One. 2013 Apr 1;8(4):e59747. doi: 10.1371/journal.pone.0059747 (PMC3613418; doi:10.1371/journal.pone.0059747)
Supplement: Appendix S1 — Estimation of species establishment probability. Table S1 The monthly temperature and precipitation under current climate and future climate. Table S2 Species establishment probability of specific species under two climate scenarios. (DOC) [file pone.0059747.s001.doc]

## Appendix

## S1 Estimation of species establishment probability

The forest gap model (LINKAGES) was used to simulate the physiological response of each species to both current and warming climate [1,2]. Individual species biomass was simulated using LINKAGES as a result of interactions of monthly climate, soil water and nutrients, and species biological traits with ecological processes. The monthly temperature and precipitation under current climate for 1961–1990 were obtained from the Northeastern Regional Meteorological Center in China, and the monthly climate under warming climate (The A2 scenario) was obtained by adding the change in temperature (°C) and precipitation (mm) between the Hadley projection for the periods 1961–1990 and 2070–2099 based on the current climate parameters.

Individual species biomass under warming climate was simulated by modifying only the LINKAGES climate file, changing monthly temperature and precipitation (Table S1). The simulated biomass was converted to the species establishment probability (Table S2) to quantify the environment suitability using an empirical method [3]:

$$e_{ij} =b_{ij} /\left\{\sqrt{\sum_{j=2}^{4} b_{ij} ,\sum_{j=2}^{4} b_{ij}^{'} }\right\}$$

Where $b_{ij}$ and $b_{ij}^{'}$ are the biomass of species $i$on land type$j$under current and warming climate respectively, $e_{ij}$ is the species establishment probability (SEP) of specie$i$on land type$j$under current climate, $\sum_{j=2}^{4} b_{ij}$ is the sum of the biomass of species$i$on all land types. $e_{ij}$ is then scaled to 0-1. $j$ starts from 2 since there are four effective land types (2–4) in our study area. Dividing by the square sum makes $e_{ij}$ comparable among different land types. The square root make $e_{ij}$ comparable by relativizing the biomass differences due to species physiological differences. The max operation ensures that $e_{ij}$ is comparable between current climate and climate warming conditions. We used the LINKAGES output from the first 10 years to examine species establishment. The output indicates that each species either completely disappears or reaches a stable trend within 10 years.

## Literature

1. Pastor J, Post WM (1988) Response of Northern Forests to Co2-Induced Climate Change. Nature 334: 55-58.

2. Post WM, Pastor J (1996) Linkages - An individual-based forest ecosystem model. Climatic Change 34: 253-261.

3. He HS, Mladenoff DJ, Crow TR (1999) Linking an ecosystem model and a landscape model to study forest species response to climate warming. Ecological Modelling 114: 213-233.

**Table S1** The monthly temperature and precipitation under current climate and future climate

| Climate scenarios | Jan. | [Feb.](javascript:void(0)) | Mar. | Apr. | May | Jun | Jul. | Aug. | Sept. | Oct. | Nov. | Dec. |
| --- | --- | --- | --- | --- | --- | --- | --- | --- | --- | --- | --- | --- |
| Monthly temperature (^◦^C) | | | | | | | | | | | | |
| Current climate | -26.94 | -23.69 | -14.41 | -1.28 | 6.13 | 12.01 | 14.81 | 11.92 | 5.98 | -4.21 | -18.46 | -24.90 |
| Future climate | -22.61 | -18.07 | -9.86 | 1.92 | 9.97 | 17.23 | 20.98 | 18.97 | 11.73 | -0.21 | -13.11 | -20.89 |
| Monthly precipitation (mm) | | | | | | | | | | | | |
| Current climate | 3.38 | 4.22 | 6.63 | 23.37 | 27.97 | 99.25 | 114.41 | 95.99 | 50.42 | 21.59 | 10.36 | 5.26 |
| Future climate | 6.45 | 10.34 | 10.27 | 28.42 | 34.32 | 133.08 | 155.92 | 110.90 | 86.46 | 26.06 | 14.54 | 9.60 |

**Table S2** Species establishment probability of specific species under two climate scenarios

| Climate scenarios | Birch | Aspen | Poplar | Willow | Larch | Spruce | Mongolian Scots pine | Dwarf pine |
| --- | --- | --- | --- | --- | --- | --- | --- | --- |
| Current climate | 0.153 | 0.010 | 0.013 | 0.018 | 0.288 | 0.060 | 0.175 | 0.225 |
| Future climate | 0.387 | 0.204 | 0.045 | 0.075 | 0.206 | 0.134 | 0.213 | 0.108 |
